# Chromosome-level assembly of the *Clinopodium gracile* genome

**DOI:** 10.3389/fpls.2024.1489102

**Published:** 2024-11-11

**Authors:** Yubang Gao

**Affiliations:** School of Life Sciences, Nanyang Normal University, Nanyang, Henan, China

**Keywords:** genome, chinese herbal medicine, Clinopodium gracile, nanopore sequence, Hi-C assembly

## Abstract

*Clinopodium gracile* is an important medicinal herb in the Lamiaceae family. This species lacks corresponding genomic resources, which significantly limits the study of its active compound synthesis pathways, breeding practices, and assessment of natural genetic variations. We assembled the chromosomal-level genome of *C. gracile* using Oxford Nanopore (ONT) technology and Hi-C sequence. The assembled genome is 307.3 Mb in size and consists of 9 chromosomes. The scaffold N50 was 36.3 Mb. The BUSCO completeness (Embryophyta_db10) of the genome was 97.2%. The genome annotates 40,083 protein coding genes. *C. gracile* and *S. miltiorrhiza* diverged approximately 30.615 million years ago. *C. gracile* has not undergone recent species-specific WGD events. A high proportion of young LTRs indicates a recent transposable element (TE) transposition burst in *C. gracile*.

## 1 Introduction

**Figure 1:**
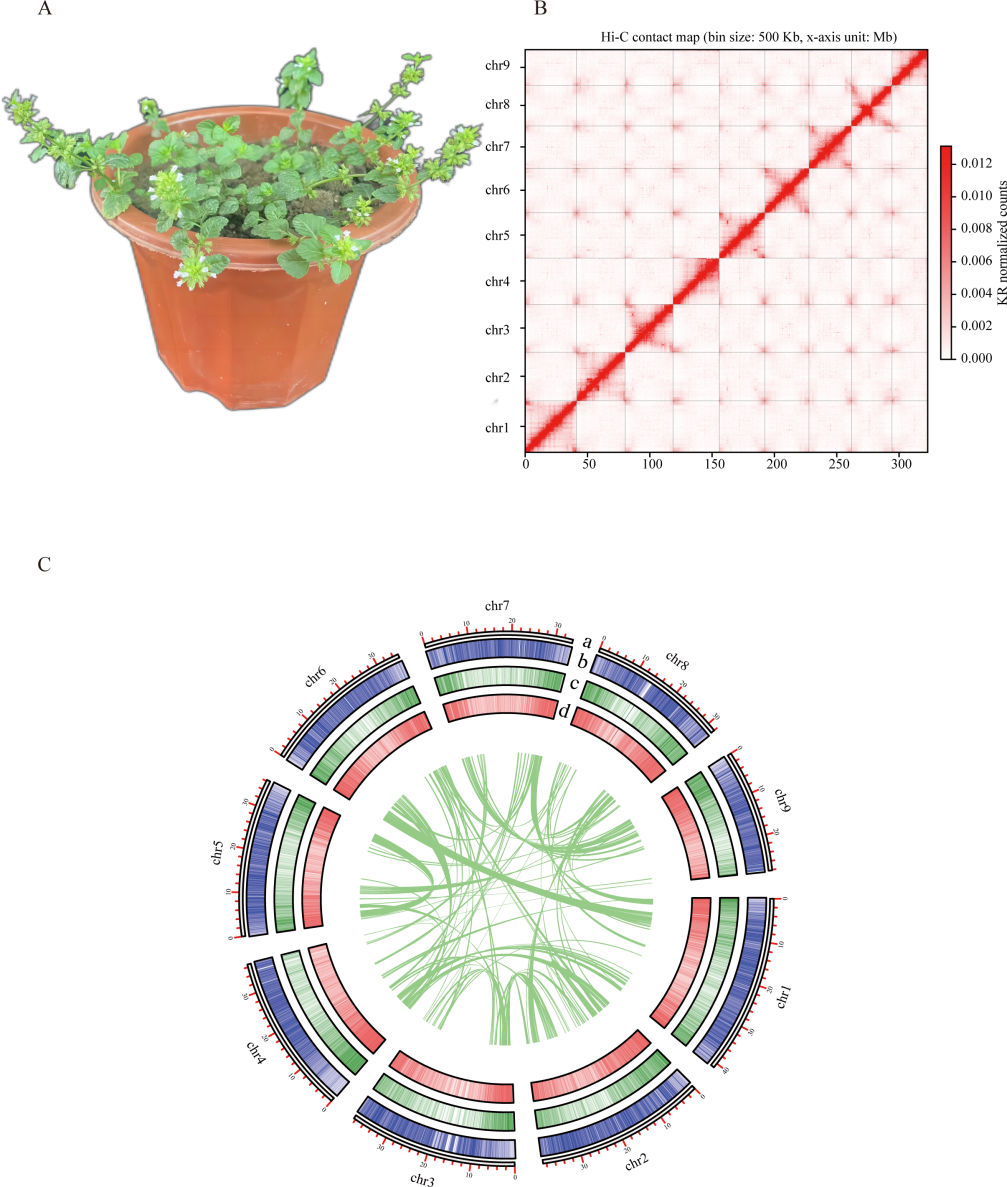
Chromosome-scale assembly of the *C*. *gracile* genome. **(A)**. The phenotype of *C*. *gracile* (The flower pot size was 15 cm). **(B)** Heat map of Hi-C interactions for the *C*. *gracile* genome. **(C)** Circos plot showing features in a 100 kb window on 9 chromosomes of the *C*. *gracile* genome. a. Length of each pseudochromosome (Mb). b. Distribution of repetitive sequences. c. Distribution of gene density. d. Distribution of the GC content.

*C. gracile* (**Figure 1A**) is a medicinal plant belonging to the genus Clinopodium in the Lamiaceae family (Dai et al., 1984). The Clinopodium genus comprises 20 species, most of which are medicinal plants (Yao et al., 2020). The triterpenoid saponins of *C. gracile* exhibit various pharmacological effects, such as anti-inflammatory (Park et al., 2010), cardioprotection (Hu et al., 2017), and anti-tumor characteristics (Dzhambazov et al., 2002). Additionally, it exhibits insecticidal activities (Chen et al., 2013). Research on *C. gracile* involves transcriptomics of different tissues analysis (Zhao et al., 2020; Shan et al., 2020). Moreover, studies on species like *Clinopodium chinese* include transcriptomics analysis (Shi et al., 2019) and microRNA analysis (Xu et al., 2022). *C. gracile* and other plants in the same genus lack corresponding reference genomes, significantly hindering the study of their active compound synthesis pathways, breeding practices, and assessment of natural genetic variations.

With continuous improvements in DNA sequencing technologies, assembling chromosome-level genomes is becoming increasingly feasible (Kong et al., 2023). Nanopore sequencing, known for its long-read capability, offers distinct advantages in genome assembly (MacKenzie and Argyropoulos, 2023). Here, we employed ONT nanopore sequencing and Hi-C sequence to assemble the *C. gracil*e genome. The assembly’s contig N50 values were 36.3 Mb. Comparative genomic analysis indicated that the *C. gracile* genome had undergone a TE insertion burst. The assembled genome with gene annotations is the first reference genome for this species and the genus. The assembled genome in this study will facilitate research on the *C. gracile* genome, metabolic engineering, and the improvement of elite cultivars.

## 2 Materials and methods

### 2.1 Material collection and genome sequencing

The plants used for genome sequencing were cultivated under laboratory conditions: 25°C, 3000 lx, and a photoperiod of 16 hours light to 8 hours dark. High molecular weight DNA (HMW DNA) was extracted for subsequent library construction. Genomic DNA was extracted using the Qiagen MagAttract HMW DNA Mini Kit, following the manufacturer’s protocol. The Hi-C libraries were prepared by chromatin crosslinking, restricted enzyme (MboI) digestion, end filling and biotinylation tagging, DNA purification and shearing. All of the prepared DNA fragments were processed into paired-end sequencing libraries. Sequencing was performed on the DNBSEQ-T7 and PromethION platforms.

### 2.2 Genome survey

We used fastp (Chen et al., 2018) version 0.20.1 to trim the raw reads. Using the trimmed data, we employed Jellyfish (Marçais and Kingsford, 2011) version 2.3.0 to calculate the K-mer distribution histogram. Genome size, heterozygosity, and repeat rate were estimated using GenomeScope 2.0 (Ranallo-Benavidez et al., 2020). Genomic ploidy was analyzed using Smudgeplot (Ranallo-Benavidez et al., 2020).

### 2.3 Genome assembly and gene annotation

The genome was assembled using NextDenovo (Hu et al., 2023) version 2.5.2. The assembled sequences were polished four times using NextPolish (Hu et al., 2020) version 1.4.1 with short read. Repetitive elements in the genome were annotated using EDTA version 2.0.1 (Ou et al., 2019). Gene prediction was conducted using Funannotate version 1.8.16 (https://github.com/nextgenusfs/funannotate), integrating *de novo* prediction, homology prediction, and transcriptome sequencing data. Functional annotation was performed using DIAMOND (Buchfink et al., 2015) version 2.0.14.152 for protein BLAST against EggNOG/SwissProt/NR/TAIR databases. tRNAs, rRNAs, miRNAs, and snRNAs were identified using Infernal (Nawrocki and Eddy, 2013) version 1.1. The completeness of the genome assembly and protein-coding genes were evaluated using BUSCO (Simão et al., 2015) version 5.2.2. The assembly quality was assessed by mapping short-read data to the assembled genome using Bowtie2 (Langmead and Salzberg, 2012) version 2.4.4. Long-read data was mapped to the assembled genome using Minimap2 (Li, 2018) version 2.24-r1122.

### 2.4 Phylogenetic analysis

Protein sequences from *C. gracile* and ten other flowering plants (*Oryza sativa*, *Amborella trichopoda*, *Arabidopsis thaliana*, *Coffea canephora*, *Theobroma cacao*, *Vitis vinifera*, *Salvia miltiorrhiza*, *Leonurus japonicus*, *Tectona grandis*, and S*olanum lycopersicum*) were utilized to create a phylogenetic tree. OrthoVenn3 (Sun et al., 2023) supported analyses of phylogenetic and gene family expansions and contractions. The process entailed using OrthoMCL (Li et al., 2003) for identifying homologous proteins and unique genes. FastTree2 (Price et al., 2010) version 2.1.7 was employed to develop the phylogenetic tree using the JTT+CAT model. SH tests verified node accuracy. Divergence among species was calculated using the r8s tool (Sanderson, 2003) version 1.81 with known divergence times between *A. thaliana* and *C. canephora*, *A. thaliana* and *V. vinifera*, *S. lycopersicum* and *T. cacao*, *S. miltiorrhiza* and *L. japonicus*. Additionally, gene family expansions and contractions were evaluated with CAFE5 (Mendes et al., 2020), applying a stochastic birth-and-death model. Assess the statistical significance via conditional likelihood, with P-values <= 0.01 indicating significant findings.

### 2.5 Duplicated gene analysis

MCScanX (Wang et al., 2012) was used to detect synteny and collinearity within and between species. Duplicated genes originating from WGDs were extracted from collinear regions. The downstream analysis script ‘duplicate_gene_classifier’ from MCScanX was utilized to categorize types of duplicated genes. Based on codon alignments using the YN substitution model, the four-fold degenerate transversion (4DTv) distances were calculated between orthologous and paralogous gene pairs within and between species. GO enrichment analysis was performed using ClusterProfiler (Wu et al., 2021) version 4.0. Circos are plotted using the TBtools (Chen et al., 2020) circos function. The link size parameter under the link region config setting was set to 0.1.

### 2.6 RNA-Seq analysis

Previously published RNA-Seq data for *C. gracile* roots, stems, leaves, and flowers (Shan et al., 2020) were downloaded. The downloaded RNA-seq reads were mapped to the *C. gracile* genome using HISAT2 (Kim et al., 2019) version 2.2.1. Gene expression levels were quantified by calculating FPKM values using StringTie2 (Kovaka et al., 2019) version 2.2.1.

## 3 Data

### 3.1 Genome assembly

32.26 Gb short-read data, 32.46 Gb Nanopore long-read data and 43.2 Gb Hi-C data were generated (**Supplementary Table S1**). Genome survey using short-read data revealed a genome size of 269.73 MB, with repeat elements constituting 36.9% and heterozygosity of 0.27% (**Supplementary Figure S1**). Genomic ploidy analysis predicts the *C. gracile* genome to be diploid (**Supplementary Figure S2**). The long-read assembly resulted in a 307 Mb genome comprising 31 contigs, with an N50 of 28.2 Mb. Post-scaffolding with Hi-C data yielded 9 pseudochromosomes with an N50 of 36.3 Mb (**Figure 1B**). The pseudochromosome ranges from 39.2 Mb to 26.9 Mb, covering 99.7% of the genome. Chromosomes were numbered in descending order of size. For the genome assembly, the BUSCO completeness was 97.21% (**Supplementary Table S2**). The mapping rates of short-read and long-read genomic data to the unmasked genome were 88.72% and 99.38%, respectively. The mapping rate of previously published RNA-Seq data (Shan et al., 2020) was 93.90%. These results indicated good assembly quality.

### 3.2 Gene prediction and gene annotation

The genome contains 51.39% repetitive sequences, with Type I transposable elements (TEs) comprising 36.35% and Type II TEs 15.04%. Within Type I TEs (LTR-RTs), the highest proportion is Gypsy (21.92%), followed by Copia (12.47%). Consistent with most plants, LTR-RT represents the most prevalent elements in the *C. gracile* genome. In *C. gracile*, the Gypsy (21.92%) and Copia (12.47%) retrotransposon families exhibit a slight contraction compared to *Salvia miltiorrhiza* (Gypsy: 29.83%, Copia: 14.77%) (Song et al., 2020).

After masking repetitive sequences, we predicted 40,083 protein-coding genes (**Figure 1C**). The average coding sequence (CDS) length of genes is 978 bp (**Supplementary Table S3**). Genes contain 4.33 exons on average. 34,193 (85.3%) predicted genes could be annotated in public databases (EggNOG, NR, Swiss-Prot, and TAIR). These results indicate that the *C. gracile* genome assembly is high quality and nearly complete. Terpene synthesis-related genes in *T. grandis* (Zhao et al., 2019) were used for homology prediction. A total of 42 genes of terpene synthesis-related pathways were predicted. Of these, 14 were involved in the mevalonate (MVA) pathway and 28 in the methylerythritol phosphate (MEP) pathway (**Supplementary Table S6**). Additionally, we predicted 732 rRNAs, 634 tRNAs, 492 miRNAs, and 1009 snRNAs.

### 3.3 Comparative genomic analysis of *C. gracile* with other plants

**Figure 2:**
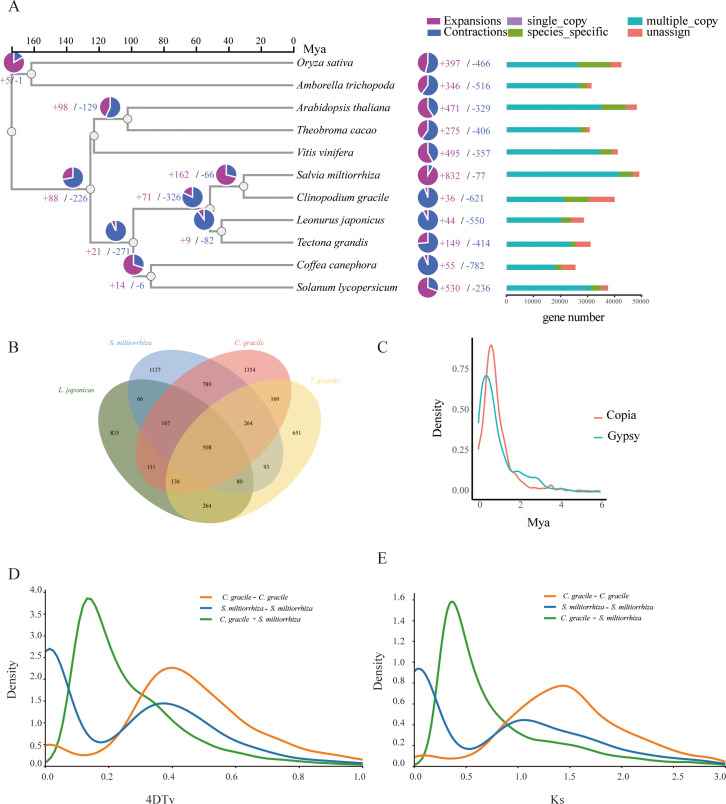
Gene family and phylogenetic tree analyses of *C*. *gracile* and other representative plant genomes. **(A)** A phylogenetic tree based on shared single-copy gene families, gene family expansions, and contractions among *C*. *gracile* and ten other species. The bar chart on the right displays gene family clustering in *C*. *gracile* and ten other plant species. **(B)** Venn Diagram Representation of Gene Family Overlaps and Specificities Among *C*. *gracile*, *L. japonicus*, *T. grandis*, and *S. miltiorrhiza* in Labiatae. **(C)** Density plot showing the burst of LTR-RTs in *C*. *gracile*. **(D)** 4DTv distribution of duplicate gene pairs in *C*. *gracile* and *S. miltiorrhiza*, calculated based on the alignment of codons with the YN substitution model. **(E)** Ks value distribution plot for orthologous gene sets between *C*. *gracile* and *S. miltiorrhiza*.

Combining the protein sequences of *C. gracile* and ten other angiosperms yielded 40,083 proteins (**Supplementary Table S4**). Clustering identified 34,011 gene families, including 319 single-copy gene families (**Supplementary Table S5**). These single-copy genes were used to construct a phylogenetic tree, incorporating known divergence times. *C. gracile* and *S. miltiorrhiza* diverged approximately 30.615 million years ago (MYA) (**Figure 2A**). Synteny analysis between *C. gracile* and *S. miltiorrhiza* revealed limited collinearity (**Supplementary Figure S3**), indicating significant genomic changes since their divergence. In *C. gracile*, there are expansions in 36 gene families and contractions in 621 gene families. The expanded genes are primarily enriched in categories related to environmental stress, such as response to growth hormone, toxic substances, and cadmium ion (**Supplementary Figure S4**). A comparison across four Lamiaceae species showed 508 conserved gene families, suggesting high conservation within the family (**Figure 2B**). *C. gracile* and *S. miltiorrhiza* have retained the most species-specific gene families, which may be functionally unique.

TEs play a significant role in genome evolution. In the *C. gracile* genome, the LTR-RT families were analyzed (**Figure 2C**). There was one peak (Gypsy or Copia) of LTR-RT amplification within the last 1 million years, indicating a recent burst in LTR-RT amplification in its genome. We identified 1,119 LTR-RTs (87.4%) with insertion times less than 2 MYA. The high proportion of young LTR-RTs suggests that TE transposition has been actively shaping the recent evolutionary history of *C. gracile*.

To further investigate the evolutionary differences between *C. gracile* and *S. miltiorrhiza*, we analyzed the four-fold degenerate transversion (4DTv) rates among orthologous gene pairs within and between species. The peak 4DTv distance of 0.17 corresponds to the speciation event that separated *C. gracile* and *S. miltiorrhiza* (**Figure 2D**). The Ks distribution plot for syntenic genes between *C. gracile* and *S. miltiorrhiza* shows similar trends (**Figure 2E**). The duplicated genes in the genome were categorized, resulting in 6,481 whole-genome duplications (WGDs), 3,738 tandem duplications, 2,529 proximal, 18,144 dispersed, and 9,722 singleton duplications. Tandem duplications are particularly enriched in the secondary metabolite biosynthetic process, response to toxic substances, and toxin metabolic process (**Supplementary Figure S5**), suggesting their role in metabolizing secondary metabolites and toxic substances. We find 225 positively selected genes (Ka/Ks > 1) and 2,022 negatively selected genes (Ka/Ks < 1) (**Supplementary Figure S6**). Positively selected genes were enriched in cellular respiration, heat acclimation, and positive regulation of auxin-mediated signaling pathways, indicating selection by harsh environmental conditions (**Supplementary Figure S7**).

## Data Availability

The datasets presented in this study can be found in online repositories. The names of the repository/repositories and accession number(s) can be found below: https://www.ncbi.nlm.nih.gov/, SRR28508814, SRR28508813, SRR29849768 https://figshare.com/, https://figshare.com/s/579e58be9da2ccbcb192.
