# Supplementary material for: Chromosome-level assembly of the Clinopodium gracile genome
Source: Front Plant Sci. 2024 Nov 11;15:1489102. doi: 10.3389/fpls.2024.1489102 (PMC11586180; doi:10.3389/fpls.2024.1489102)
Supplement: Supplementary file 2 [file Image1.pdf]

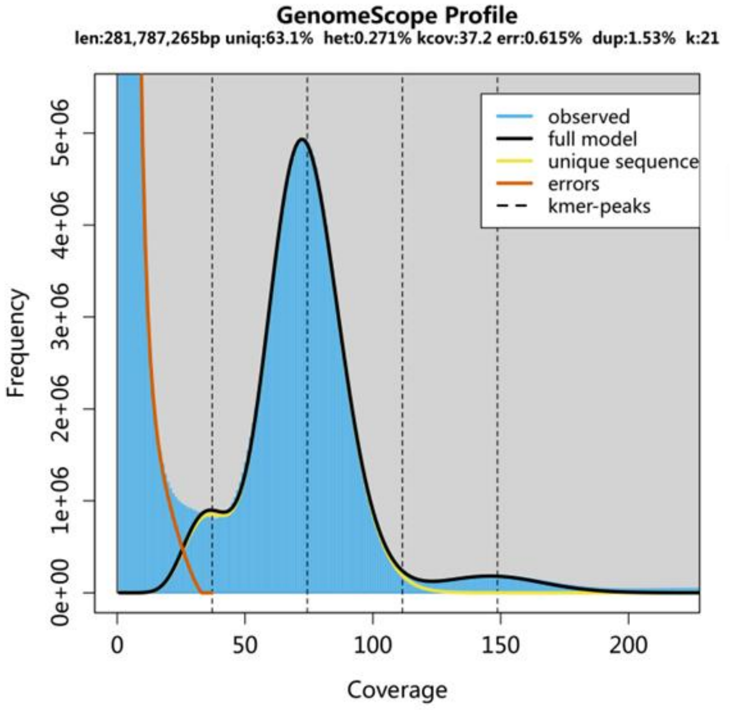

Fig. S1 Genome survey of *C. gracile*.

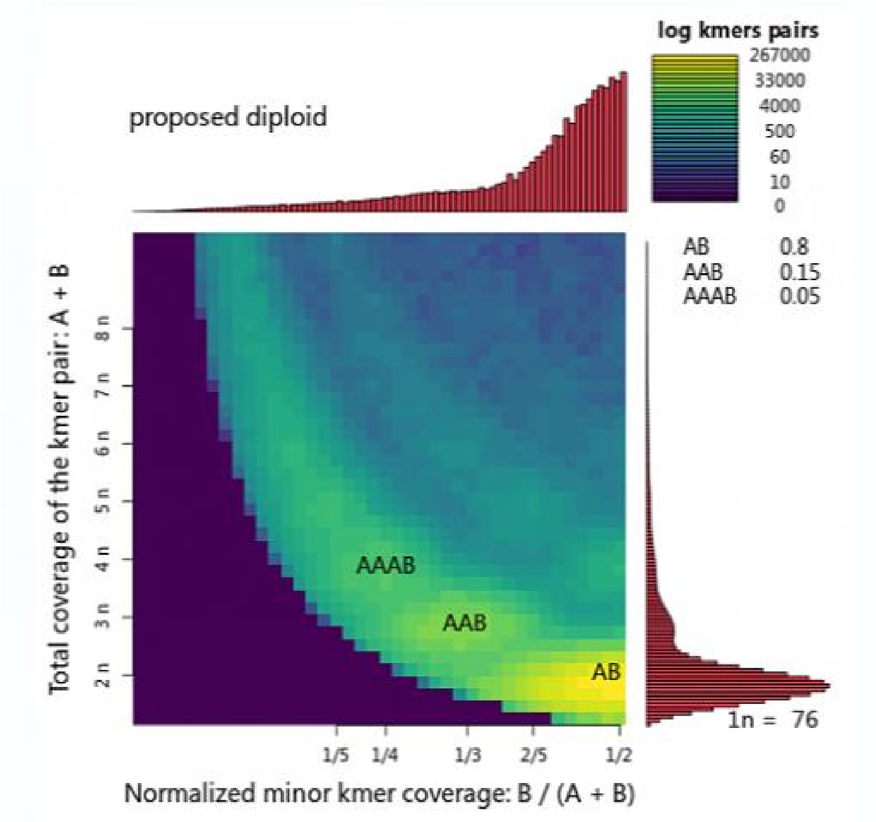

Fig. S2 Genome ploidy analysis of *C. gracile*.

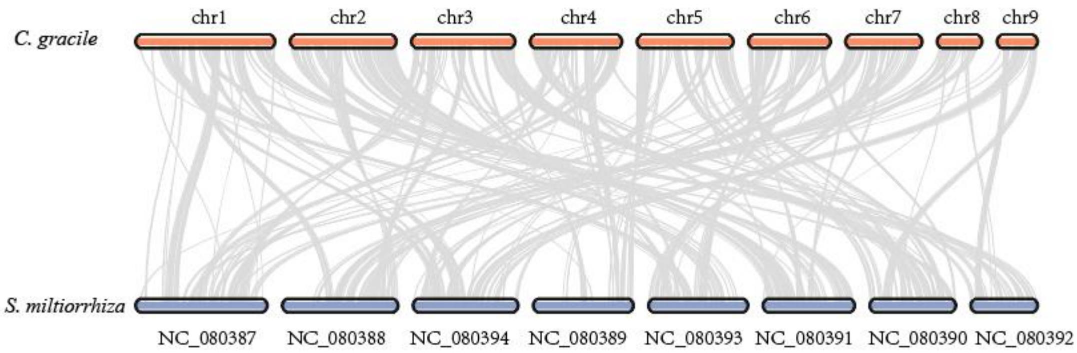

Fig. S3 Colinearity analysis of *C. gracile* and *S. miltiorrhiza.*

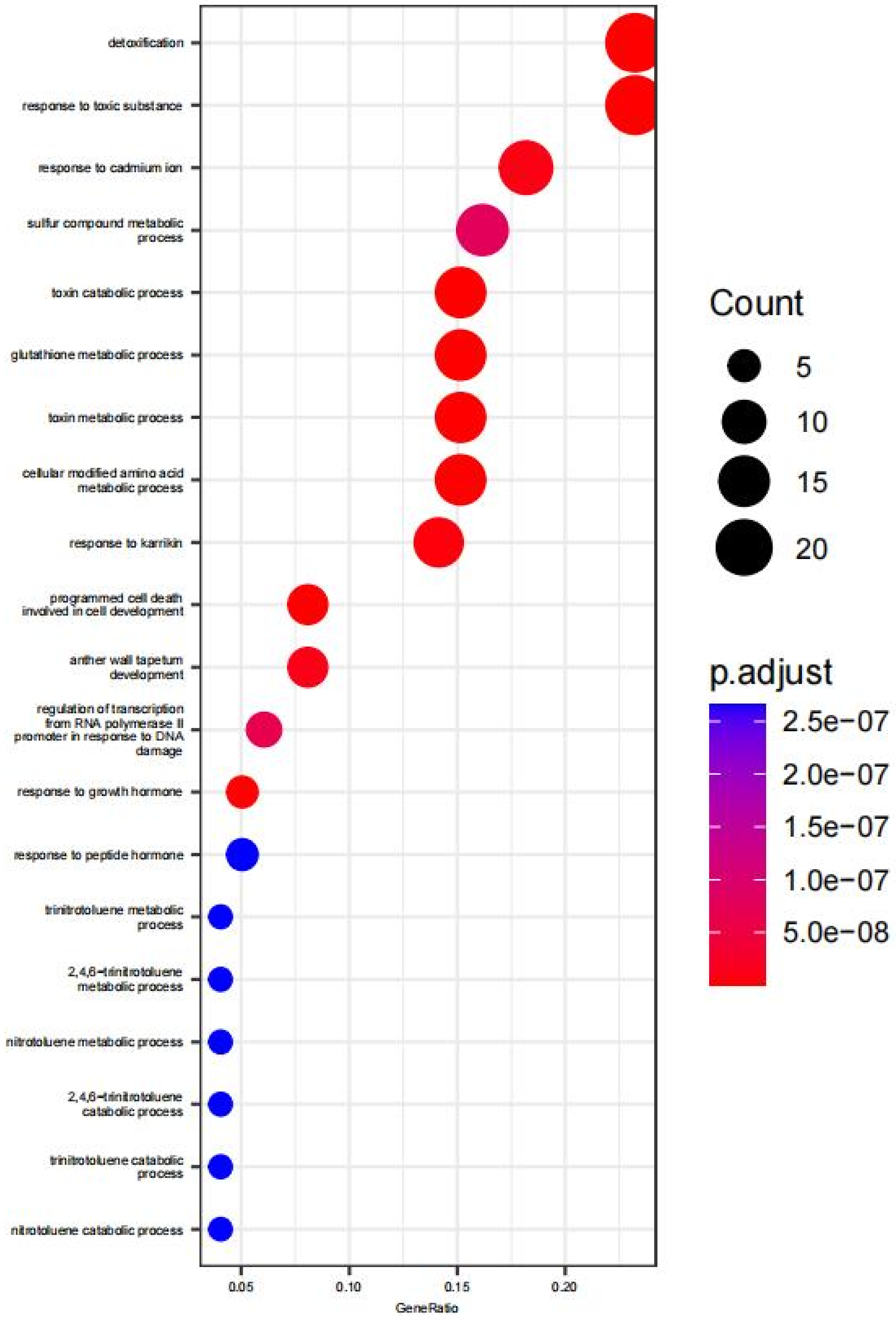

Fig. S4 GO enrichment Analysis of expansion genes.

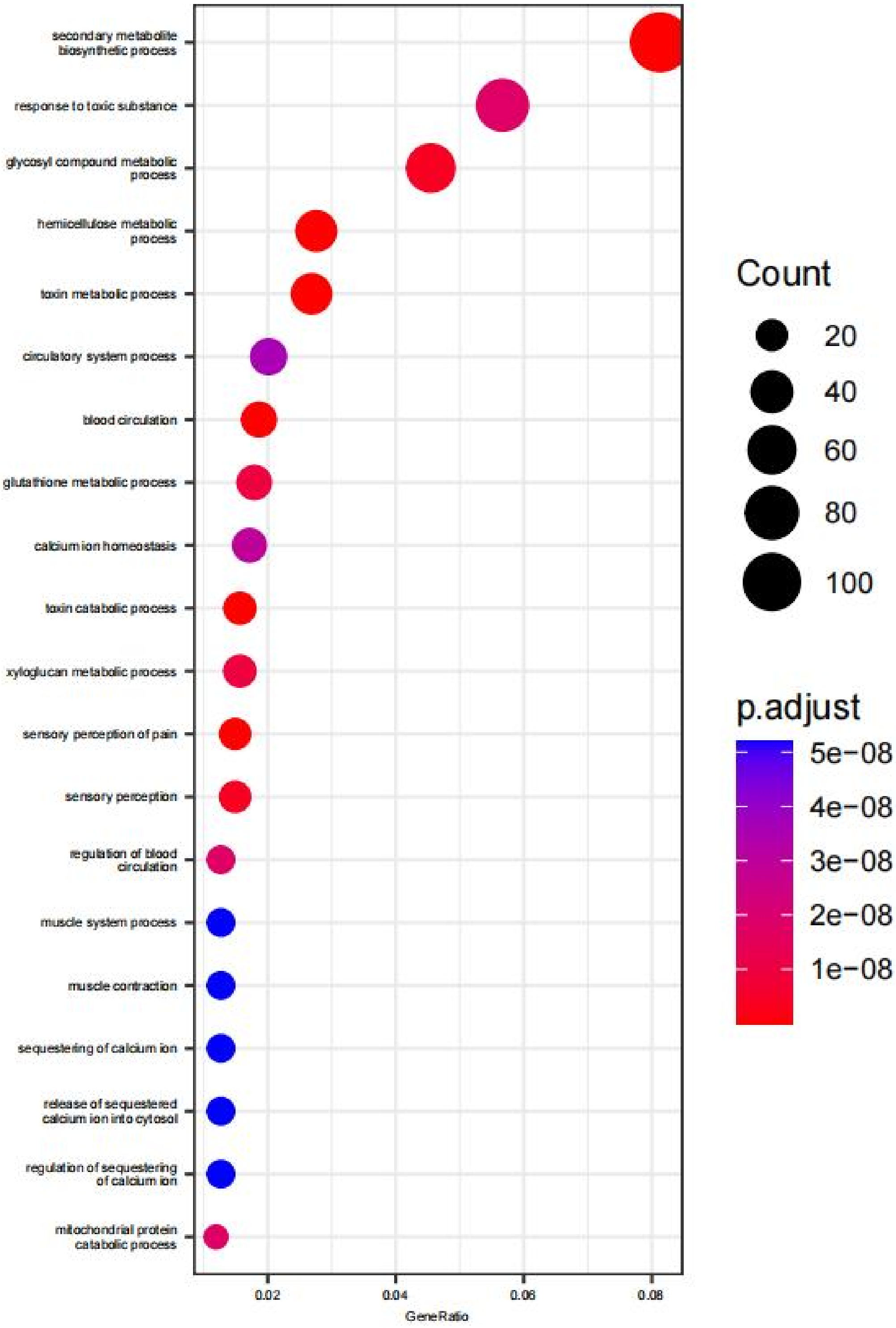

Fig. S5 GO enrichment Analysis of tandem duplicated genes.

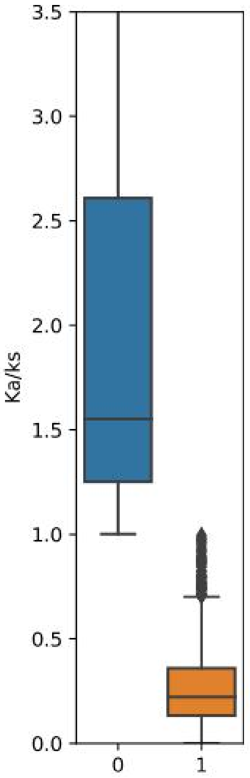

Fig. S6 Ka/Ks analysis.

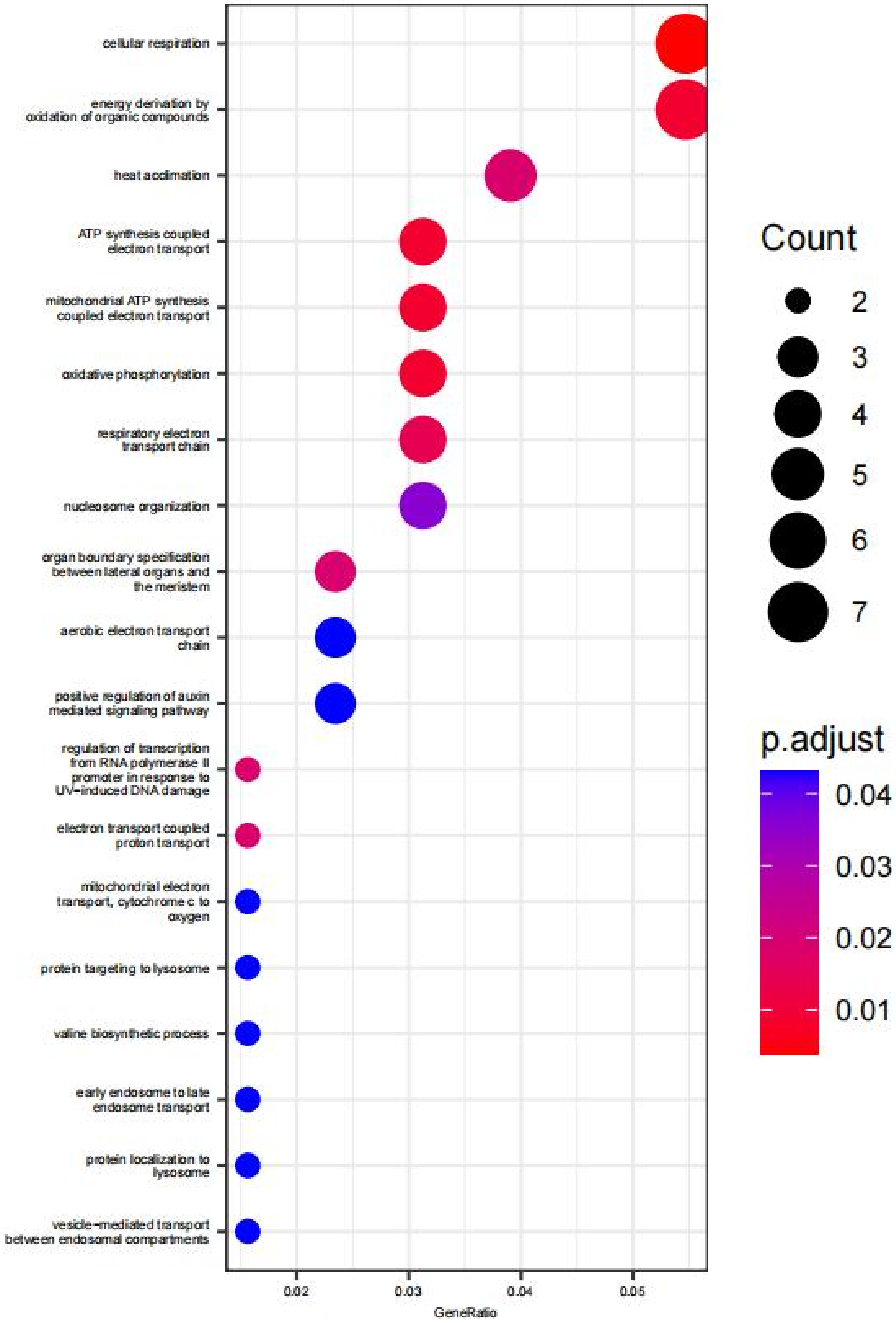

Fig. S7 GO enrichment Analysis of positively selected genes.
